# Vascularization of the human intervertebral disc: A scoping review

**DOI:** 10.1002/jsp2.1123

**Published:** 2020-09-15

**Authors:** Dale E. Fournier, Patti K. Kiser, J. Kevin Shoemaker, Michele C. Battié, Cheryle A. Séguin

**Affiliations:** ^1^ Health and Rehabilitation Sciences (Physical Therapy), Faculty of Health Sciences The University of Western Ontario London Ontario Canada; ^2^ Bone and Joint Institute The University of Western Ontario London Ontario Canada; ^3^ Department of Pathology and Laboratory Medicine, Schulich School of Medicine & Dentistry The University of Western Ontario London Ontario Canada; ^4^ School of Kinesiology, Faculty of Health Sciences The University of Western Ontario London Ontario Canada; ^5^ Department of Physiology and Pharmacology, Schulich School of Medicine & Dentistry The University of Western Ontario London Ontario Canada; ^6^ School of Physical Therapy, Faculty of Health Sciences The University of Western Ontario London Ontario Canada

**Keywords:** annulus fibrosus, anulus fibrosus, blood supply, blood vessel, cartilage endplate, intervertebral disc, intervertebral disk, nucleus pulposus, vascularisation, vasculature

## Abstract

Intervertebral discs (IVDs) are often referred to as the largest avascular structures of the human body, yet a collective resource characterizing the vascularization of the IVD does not exist. To address this gap, the objective of this study was to conduct a comprehensive search of the literature to review and summarize current knowledge of the prevalence and localization of blood supply in human IVDs, with a scoping review. A comprehensive search of peer‐reviewed publications on the topic of IVD vascularization in humans was conducted across six electronic databases: PubMed, EMBASE, MEDLINE, Scopus, Web of Science, and BIOSIS Previews. Studies of humans were included regardless of age, sex, ethnicity, and health status, with the exception of IVD herniation. Two independent reviewers screened titles and abstracts and full‐texts according to eligibility criteria. The review was conducted and reported according to Preferred Reporting Items for Systematic Reviews Extension for Scoping Reviews guidelines. Our search yielded 3122 articles, with 22 articles meeting the inclusion criteria. The study samples ranged in age from fetal to >90 years and included both sexes, various health statuses, and used different methodologies (eg, histology, medical imaging, and gross dissection) to assess vasculature. Overall, consistent observations were that (a) the nucleus pulposus of the IVD is avascular throughout life, (b) both the cartilage endplates and annulus fibrosus receive considerable blood supply early in life that diminishes over the lifespan, and (c) vascular ingrowth into the cartilage endplates and inner layers of the annulus fibrosus is commonly associated with damaged or disrupted tissue, irrespective of age. Histology and immunohistochemistry are often used to report vascularization of the IVD. The body of the current literature suggests that the IVD should not be generalized as an avascular tissue. Instead, vascularization of the IVD differs based on the constituent tissues, their age, and state of degeneration or damage.

## Graphical abstract

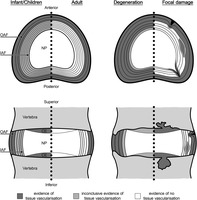

## 1 INTRODUCTION

Human intervertebral discs (IVD) are complex fibrocartilaginous joints that are generally referred to as the largest avascular structures in the human body. IVDs are composed of three distinct but interdependent tissues. The nucleus pulposus is the innermost gelatinous tissue composed primarily of proteoglycans and water.^1^ The nucleus pulposus has been shown to naturally inhibit endothelial cell migration through the secretion of an anti‐angiogenic extracellular matrix composed of proteoglycans (eg, aggrecan) and sulfated glycosaminoglycans (eg, chondroitin sulfate).^2^, ^3^ The cartilage endplates are thin layers of hyaline cartilage that anchor the IVD to the adjacent vertebral bones and act as a selectively permeable barrier for diffusion of nutrients into the IVD.^4^ The annulus fibrosus is a series of well‐organized concentric lamellae of fibrocartilage that encompass the nucleus pulposus of the IVD.^5^, ^6^

Although the IVD is often described in general terms in the literature as being avascular, previous studies have reported blood vessels confined to the outer one‐third of the annulus fibrosus.^7^, ^8^ Moreover, there is controversy regarding the specific localization of blood vessels within the IVD, most often associated with degeneration. These contradictory claims may be associated with divergent criteria and/or specificity used to identify regions of the IVD. Based on the methodologies used, the term “nucleus pulposus” can be used to indicate whatever tissue is found in the center of an IVD, or it can be defined as the histologically distinct tissue (comprising proteoglycans, type II collagen, and chondrocyte‐like cells) found near the center of the IVD. The critical difference is that histologically distinct annulus fibrosus fibrocartilage can be found collapsed into the central region of a decompressed or severely degenerated IVD, and this tissue can contain blood vessels.^9^ To date, a comprehensive overview of the current literature that characterizes the prevalence and localization of blood supply of the human IVD across the lifespan has not been conducted. Addressing this gap in our understanding of IVD vascularization has relevance to IVD biology, pathobiology, and development of regenerative strategies.

The primary pathway for nutritional supply to IVD cells is through passive diffusion from the richly vascularized vertebral bony endplate through the cartilage endplate.^4^ Previous studies have shown oxygen concentration and pH differences of the IVD, with low oxygen and pH levels in the center of the nucleus pulposus.^4^ Further, oxygen and glucose concentrations are higher in the annulus fibrosus compared to the nucleus pulposus.^4^ Of note, the gradient of oxygen and glucose concentrations across the IVD corresponds to a gradient of cell density, which is higher in the annulus fibrosus and lower in the nucleus pulposus.^4^ This gradient leads to differences in cellular demand, which is dictated by the available supply of nutrients.^4^ However, the extent of human IVD vascularization throughout life and the source of blood vessels directly supplying the IVD is currently unclear. Overall, an improved understanding of the vascularization of the IVD associated with physiological aging may further inform the mechanisms of nutrient delivery, which is relevant to better understanding age‐related changes impacting cell survival, tissue repair, and IVD degeneration ultimately leading to back pain.^10^

The association between vascular ingrowth and IVD disease status in humans is poorly understood. Clinical studies are limited to small sample sizes from severely diseased tissue retrieved from surgery and postmortem evaluations. Surgical tissues are often restricted to fragments of resected tissue, most commonly from symptomatic herniated IVDs. Moreover, postmortem tissues are often associated with advanced age and confounded by unknown comorbidities of donors. This has prevented a detailed characterization of the prevalence and spatial localization of blood vessels of the IVD, under nonpathological and pathological conditions.

### 1.1 Research question

The overarching objective of this scoping review is to summarize the available literature related to vascularization of the human IVD. An a priori search of the current literature was conducted in May 2019 across PubMed, BMJ Open, Joanna Briggs Institute Database and no published or ongoing reviews on the topic were identified. Specific sub‐questions included: (a) which methods and techniques are most effective to report vascularization of the IVD in humans? (b) does vascularization of the IVD differ across the lifespan? and (c) are changes in vascularization associated with IVD degeneration?

## 2 MATERIALS AND METHODS

The scoping review was organized according to the six‐step framework originally proposed by Arksey and O'Malley,^11^ enhanced by Levac et al,^12^ and more recently refined by Peters et al and The Joanna Briggs Institute.^13^, ^14^ Scoping reviews utilize similar methods as systematic reviews to identify and assess the available literature; however, scoping reviews target boarder research questions than systematic reviews. To maintain the quality and integrity of the scoping review, it was also conducted in accordance with the Preferred Reporting Items for Systematic Reviews Extension for Scoping Reviews (PRISMA‐ScR).^15^

### 2.1 Eligibility criteria

This scoping review included only peer‐reviewed articles that described the vascularization of the IVD in a human population. No restrictions were set based on age, sex, ethnicity, or health status (except for IVD herniation) in order to capture any descriptions of vascular structures within the IVD. Studies focused on IVD herniation were exclude since previous histological analysis of herniated IVD tissues from the lumbar spine has shown the invasion of small blood vessels,^16^ which is likely the result of an extradiscal inflammatory response to the extruded material. Moreover, this scoping review considered articles that evaluated vascularization of the IVD through a variety of different methodologies including histology, gross dissection, and medical imaging. Importantly, the outcomes of these articles had to address the presence of vasculature in the IVD and describe its localization within the tissue. We integrated consultation with experts in the fields of vascularization and microanatomy throughout this scoping review to ensure the appropriateness of search terms and applicable methodologies as well as the accuracy of the scoping review results and interpretation of the data.

### 2.2 Information sources

Quantitative and descriptive articles from peer‐reviewed journals were considered for inclusion. Narrative reviews, letters, and editorials were screened to ensure that original sources were included. No restrictions were set regarding language or year of publication. Six key electronic databases were searched: PubMed (1966‐), EMBASE (1947‐), MEDLINE (1946‐), Scopus (1966‐), Web of Science (1900‐), and BIOSIS Previews (1926‐). These databases include a broad range of the literature pertaining to biomedicine, health, life and physical sciences.

### 2.3 Search strategy

A systematic three‐step search strategy was followed based on the framework outlined by the Joanna Briggs Institute,^14^ and implemented through consultations with experienced health sciences librarians at our institution. First, an initial search was performed in PubMed to identify seminal articles related to the review question. This preliminary search was followed by a comparable search in EMBASE, to explore words in the titles and abstracts, indexing nomenclature, keywords, and various spelling associated with our search topics of humans, IVD, and vasculature. Second, additional indexing terms and keywords were retrieved from seminal articles to form the comprehensive search strategy, which was again reviewed by experienced librarians for potential sources of error. This comprehensive search strategy was then modified to fit the requirements of each included information source. As per PRISMA‐ScR guidelines, an example of a comprehensive search for one of the information sources can be found in Appendix S1.^15^ Lastly, the reference lists of articles meeting full‐text inclusion were screened for additional relevant articles that may have been missed in our search.

### 2.4 Selection of sources of evidence

All retrieved article citations were imported into the reference manager software package to remove duplicates and distribute to secondary evaluators (EndNote VX.9, Clarivate Analytics: PA). A slight modification to the traditional two‐step approach of reviewing titles and abstracts and then full‐texts was used.^11^ First, all titles and abstracts (n = 3122) were screened by one reviewer to exclude irrelevant articles, according to the following criteria: (a) animal studies; (b) topic not IVD; and (c) studies focused on surgical applications. Surgically oriented articles which focused on surgical techniques, medical devices, and case reports of clinical complications during surgery were excluded as they may describe the general anatomy but do not specifically characterize the prevalence or localization of the vascularization of the IVD. Second, screening of the remaining list of titles and abstracts (n = 718) was conducted independently by two reviewers to minimize selection bias. In addition to the criteria used in the initial screening, articles were excluded if the topic of the study was solely herniated IVD tissue without controls or characterized vasculature of vertebra but not the IVD. Articles were categorized as “include, exclude, or uncertain,” and the screening results were compared between the two reviewers. During a consensus meeting, the uncertain articles and any other disagreements were resolved by discussion. Third, the articles that met the inclusion criteria based on titles and abstracts were retrieved and assessed for full‐text eligibility by two independent reviewers, and disagreements were resolved during another consensus meeting. At the end of analysis, the comprehensive search was rerun to identify articles published during the review process. Two independent reviewers screened these titles and abstracts using the same eligibility criteria and results were compared.

### 2.5 Data items and charting

Data were aggregated from all full‐text articles included. An electronic data extraction chart was created to capture general citation information, as well as information on the study population (eg, source of tissue, age, sex, health status) and vascularization measurements (eg, prevalence and localization) related to the research question.^14^, ^15^ The data extraction chart was tested by two reviewers, using three randomly selected included articles, before one reviewer independently assessed and charted the remaining included articles. The data extraction chart was modified and revised as necessary, during the iterative process of data extraction.

### 2.6 Synthesis of results

Articles were grouped by anatomical features, such as the region of the vertebral column or the specific IVD tissue studied. Articles were also grouped by the methodology used to assess vasculature and, when possible, by the characteristics of the study population (eg, age and health status).

## 3 RESULTS

### 3.1 Selection of sources of evidence

**FIGURE 1:**
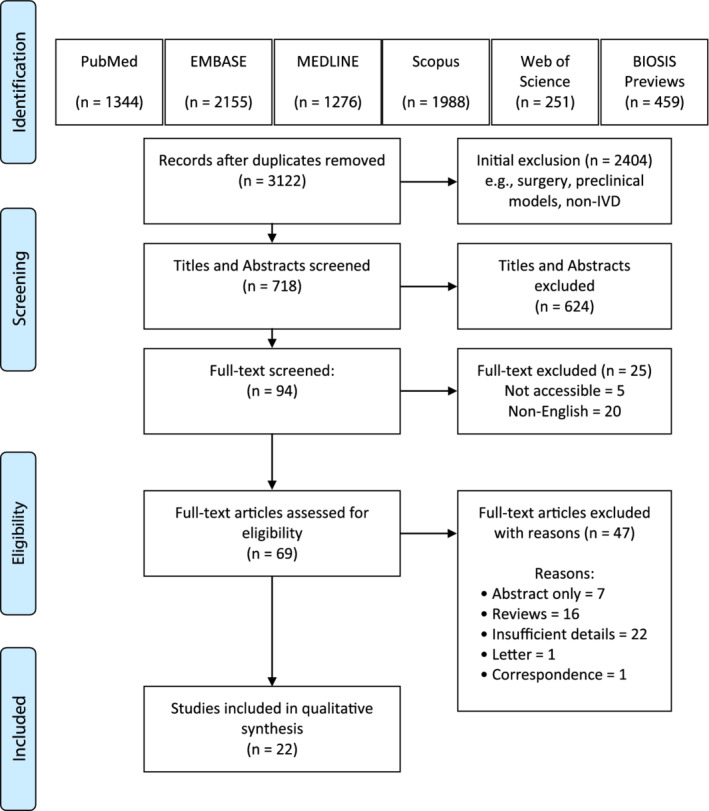
PRISMA flow diagram of the article selection process of information sources

After duplicates were removed, a total of 3122 articles were identified from our comprehensive search, of which 2404 were clearly irrelevant to the research question and excluded during preliminary screening. Of the remaining 718 articles, another 624 articles were excluded following independent screening of their titles and abstracts by two reviewers, leaving 94 articles to be retrieved for full‐text review to determine eligibility. Using IBM SPSS Statistics (Version 26.0, Armonk, NY), a Cohen's kappa statistic of 0.55 was found between reviewers. Landis and Koch suggest that Kappa statistics from 0.41 to 0.60 indicate moderate agreement.^17^ Based on our study design, all discrepancies were discussed during a consensus meeting to ensure eligible articles were included in the analysis. Of the full‐texts evaluated, 20 were published in a non‐English language and were not able to be translated; 5 were unavailable; and 47 were excluded following full‐text review. Ultimately, 22 articles were considered eligible for this scoping review (Figure 1).

The comprehensive search was rerun on 06 April 2020 to ensure recently published articles during screening and analysis were not missed. A total of 130 articles were captured in this search and the titles and abstracts were independently screened by two reviewers. No new articles were considered eligible for inclusion.

### 3.2 Results of individual sources of evidence

The general citation information of the included articles, study sample, and methodology used are presented in Table 1. The articles included were either descriptive (45%, n = 10/22) or case‐control study designs (55%, n = 12/22). Typically, case‐control studies compared IVD tissues retrieved from surgery to postmortem tissues without a known medical history of back pain, radiculopathy, or myelopathy. For several of the articles, only the specimens from study's control group met the eligibility criteria for inclusion for this scoping review.

**TABLE 1:** Details of included articles: information and study sample characteristics

Citation information				Study sample						
First author	Year	Region	Study design and method	Tissue	Region of spine	Donors	Female	Male	Age	Health‐status
Eckert^18^	1947	USA	Case–control^a^ ^,^ ^b^	*s*	*L4‐S1*	*166*	*66*	*100*	*13‐66*	*IVD herniation*
				pm	L5‐S1	40	–	–	0‐69	“normal backs”
Hirsch^19^	1953	SWE	Descriptive^a^ ^,^ ^b^ ^,^ ^c^	pm	Lumbar	120	–	–	0.5‐90	–
Hirsch^20^	1967	SWE	Descriptive^a^ ^,^ ^c^	pm	Cervical	111	61	50	0–96	No spinal disorders
Hassler^21^	1969	SWE	Descriptive^a^ ^,^ ^b^	pm	C5‐T1, L3‐S1	28	–	–	fetal‐82	Not reported, except for 1 donor with spondylosis
Donisch^22^	1971	CAN	Descriptive^a^ ^,^ ^b^ ^,^ ^c^	pm	L4 IVD	68	–	–	0–11	No spinal disorders
Jamiolkowska^23^	1981	POL	Descriptive^b^	pm	Vertebral column	16	–	10	fetal‐77	–
Rudert^24^	1993	DEU	Descriptive^a^	pm	L2‐4	24	12	12	fetal‐90	–
Kauppila^7^	1995	FIN	Descriptive^a^ ^,^ ^b^ ^,^ ^c^	pm	L1‐S1	22	8	14	20‐73	No spinal disorders
Stabler^25^	1996	DEU	Case–control^a^ ^,^ ^c^	s	T9‐S1	53	–	–	31‐72	Painful spine syndrome
				c		34	–	–	–	No spinal disorders
Chandraraj^26^	1998	AUS	Descriptive^a^	pm	L2‐4	20	–	–	0.5‐22	–
Repanti^27^	1998	GRC	Case–control^a^	*s*	*L3‐S1*	*84*	*–*	*–*	*24‐60*	*IVD herniation*
				pm	L1‐S1	24	–	–	fetal‐80	No IVD herniation
Pai^28^	1999	IND	Case–control^a^	*s*	*C5‐6; T7‐8; L1‐S1*	*75*	*15*	*60*	*11‐68*	*IVD prolapse*
				pm	C6‐7, T7‐8, L3‐S1	15	–	‐	adults	No IVD prolapse
Johnson^29^	2001	GBR	Descriptive^a^	s	L2‐S1	15	8	7	14‐50	Low back pain, IVD degeneration
Boos^30^	2002	CHE	Case–control^a^ ^,^ ^b^ ^,^ ^c^	s	L4‐S1	13	3	10	14‐68	Spinal interventions
				pm	L1‐S1	44	15	29	fetal‐88	No spinal disorders
Melrose^31^	2003	AUS	Descriptive^a^	pm	Vertebral column	6	–	–	fetal	–
Johnson^32^	2007	GBR	Case–control^a^	s	Lumbar IVDs	21	15	8	11‐62	Pathological conditions
				s		2			21	Fracture or dislocation
Nerlich^8^	2007	DEU	Case‐control^a^	s	T11‐S1	20	–	–	–	IVD degeneration
				pm	L1‐S1	42	19	23	0–86	No spinal disorders
Kokubo^33^	2008	JPN	Case–control^a^ ^,^ ^c^	s	C3‐T1	198	77	121	25‐78	IVD degeneration
				s	C3‐T1	166	61	105	32‐83	Spondylosis
				pm	Cervical	4	–	–	68‐78	No symptoms
Karamouzian^34^	2010	IRN	Case–control^a^ ^,^ ^c^	*s*	*L4‐S1*	*90*	*31*	*59*	*17‐50*	*IVD herniation*
				pm	L5‐S1	60	19	41	19–50	No spinal disorders
Lama^35^	2013	GBR	Case–control^a^ ^,^ ^c^	*s*	*L2‐S1*	*21*	*13*	*8*	*35‐74*	*IVD herniation*
				s	L2‐S1	11	5	6	39‐72	IVD degeneration
Binch^36^	2015	GBR	Case–control^a^	s	C3‐4, 5‐7; L3‐S1	61	–	–	–	Nerve compression
				pm	L3‐S1	22	–	–	–	–
Lama^9^	2018	CAN	Case–control^a^ ^,^ ^c^	*s*	*L2‐S1*	*10*	*8*	*2*	*37‐68*	*IVD herniation (pain)*
				*s*	*L3‐S1*	*11*	*5*	*6*	*35–74*	*IVD herniation (no pain)*
				s	L2‐S1	11	6	5	39–72	IVD degeneration
				s	T12‐L5	8	8	0	14–15	Scoliosis

*Note:* Citation information: First author listed, year of publication, location displayed as 3‐letter country abbreviation, based on corresponding author's information. Study samples: Italicized are sample groups that did not meet the scoping reviews eligibility criteria; however, the comparison group did. Tissues retrieved from c, controls; pm, post‐mortem; or s, surgery. Region describes the anatomical region of the spine studied: C, cervical; T, thoracic; L, lumbar; and S, sacral. Precise motion segments are displayed with original authors described. Age displayed in years and presented as ranges. '–' represents when information was not provided by the original authors.

Abbreviation: IVD, intervertebral disc.

^a^ Methodology used in study design is histology.

^b^ Methodology used in study design is gross evaluation.

^c^ Methodology used in study design is imaging.

#### 3.2.1 Study samples

Of the 22 studies included, 10 (45%) had a total sample size of <50 donors, 6 (27%) had 50 to 100 donors, and the remaining 6 (27%) had >100 donors. Individual group sample sizes ranged from 4 to 198 donors (median = 24). Most studies examined more than one spinal motion segment (IVD and the adjacent vertebra on either side) from each donor. The age of the study samples ranged from fetal/stillborn to greater than 90 years of age. While most studies reported the age range of donors; in studies that did not report age or used nonspecific descriptors, data were not assumed to represent any specific age group. In general, there were more male donors than female. However, the sex of the donors was reported in only half of the articles from which data were extracted. As a result of the variability in the annotation of results, sex differences in vasculature of the IVD could not be discerned in this scoping review. Additionally, none of the studies reported specific differences attributed to biological sex. Instead, sex was reported with the characteristics of the study samples and was presumed to be insignificant with respect to vascularization of the IVD.

IVD tissues were retrieved from either surgery or postmortem autopsy. Surgical tissues were retrieved from operations to alleviate symptoms associated with IVD herniation, prolapse, nerve root compression, or degeneration. Data from surgical samples of IVD herniation or prolapse were not included in the current scoping review. Importantly, the original authors' main inclusion criterion for control or cadaveric tissues was often the absence of pathology (eg, no IVD herniation) or a “normal” appearing spine. However, since medical histories related to spinal health were not always available, control and postmortem samples may not always be representative of “healthy” IVD tissue. Postmortem tissues were obtained from autopsy for a variety of reasons, such as terminated pregnancy or stillbirth, and causes of acute death (eg, motor‐vehicle accident). The original authors' exclusion criteria for postmortem tissues were malignant disease and a history of back pain or spine surgery when medical records were available.

With respect to the anatomical region of the vertebral column studied, 2/22 articles included only the cervical region and 14/22 included only the lumbar region. The remaining 6/22 articles reported on IVDs from multiple spinal regions. In fetal and stillborn samples, IVDs from all regions of the vertebral column were examined.

#### 3.2.2 Methodologies

The methods used to study the IVD included histology (n = 21/22), medical imaging with or without contrast agent (n = 11/22), and gross evaluation (n = 6/22). Tissue sections were typically acquired in the sagittal or transverse planes and stained with hematoxylin and eosin for histopathological evaluation and complemented by immunohistochemistry for endothelial or smooth muscle cells to highlight features of blood vessels (eg, CD31, CD34, VEGF). Histological characteristics of blood vessels ranged from single‐layer endothelial capillaries to arterioles with smooth muscle walls. Three studies used radiography with a contrast medium to evaluate vasculature of the IVD.^7^, ^20^, ^21^ One study utilized a vascular perfusion method of India ink and gelatin to visualize blood vessels of the IVD with dissection.^23^

### 3.3 Synthesis of results

**FIGURE 2:**
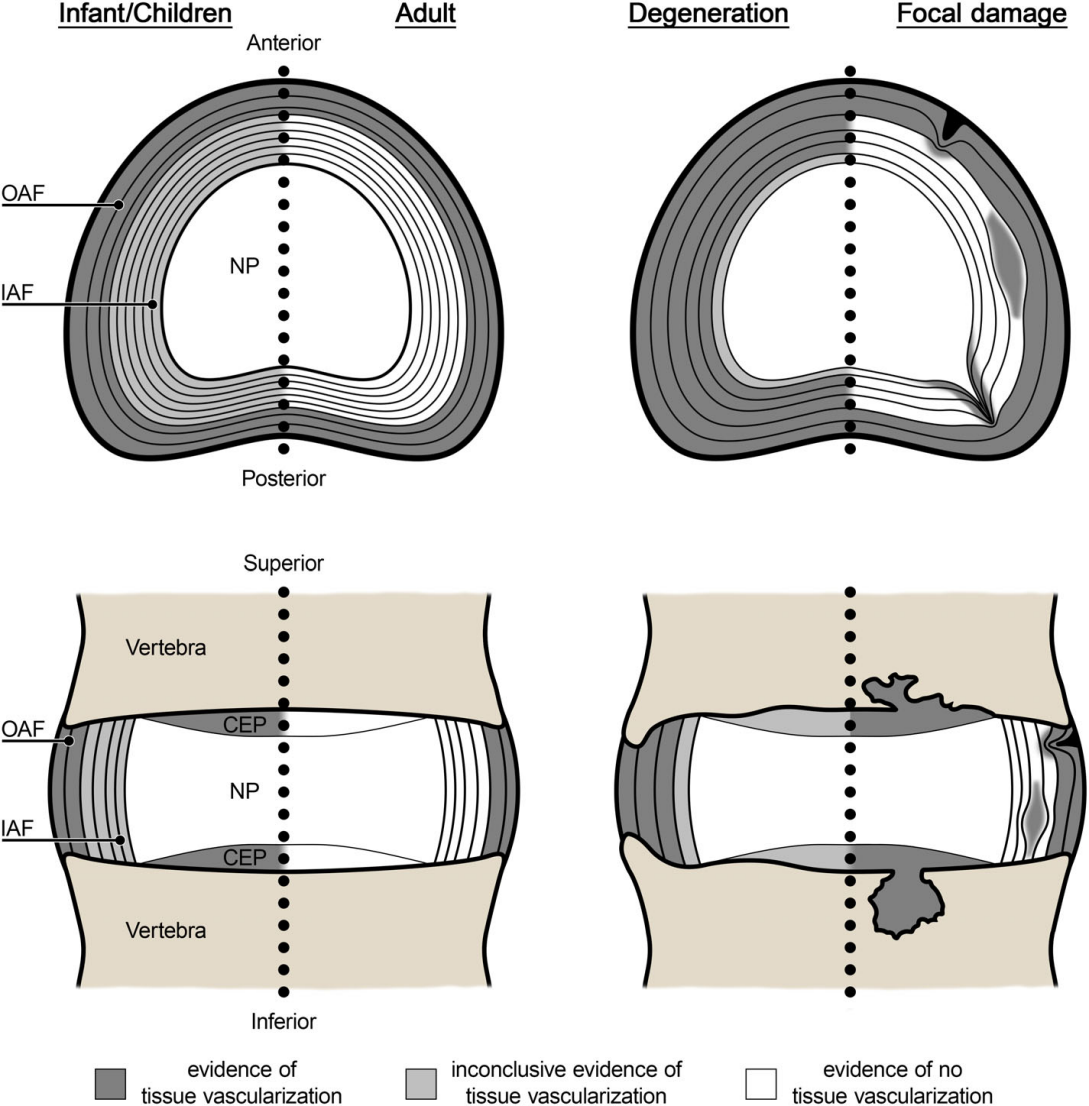
Schematic summarizing the vascularization of human intervertebral disc. Illustrations are presented across ages, with degeneration, and focal damage. The top row is a transverse or horizontal section and the bottom row is a coronal or frontal section of the intervertebral disc. Within the schematics, the level of evidence of tissue vascularization is represented by dark gray, evidence of tissue vascularization; light gray, inconclusive evidence of tissue vascularization; and white, evidence of no tissue vascularization. In the focal damage schematics, various forms of intervertebral disc damage are displayed (peripheral, circumferential, and radiating tears of the annulus fibrosus, and cartilage endplate damage). Schematic and anatomical features are not drawn to scale. CEP, cartilage endplate; IAF, inner annulus fibrosus; NP, nucleus pulposus; OAF, outer annulus fibrosus

The primary findings from each included article have been grouped based on the three interdependent tissue types of the IVD in Tables 2, 3, 4 and summarized in a schematic in Figure 2. As a result of inconsistencies in the annotation of sex and the preferential focus on lumbar spine, differences based on sex or anatomical region could not be elucidated in the current scoping review.

**TABLE 2:** Vascularization of the nucleus pulposus

Citation		Nucleus pulposus			
Author	Year	Fetal or infant (0‐2)	Children and youth (2‐25)	Adult (25‐64)	Older adult (65+)
Hirsch^19^	1953	• Avascular throughout the lifespan			
Hirsch^20^	1967	• Avascular throughout the lifespan			
Donisch^22^	1971	• Blood vessels through cartilage endplate that do not penetrate into NP		—	—
Jamiolkowska^23^	1981	• Avascular throughout the lifespan			
Rudert^24^	1993	• Avascular throughout the lifespan			
Stabler^25^	1996	—	—	• No enhancement or vascularization in patients or controls	
Chandraraj^26^	1998	• Blood vessels through cartilage endplate that do not penetrate into NP		—	—
Repanti^27^	1998	• Avascular throughout the lifespan			
Pai^28^	1999	—	—	• Blood vessels never present	
Johnson^29^	2001	–	• No nerves or blood vessels throughout the lifespan		—
Nerlich^8^	2007	• Avascular throughout the lifespan			
Karamouzian^34^	2010	—	• Microscopic angiogenesis observed in 4/60 controls		—
Lama^35^	2013	—	—	• Blood vessels never present	
Binch^36^	2015	• Blood vessels detected at NP‐AF boundary with degeneration			
Lama^9^	2018	—	• No blood vessels in scoliosis	• Blood vessels never present	

Abbreviations: AF, annulus fibrosus; NP, nucleus pulposus.

**TABLE 3:** Vascularization of the cartilage endplate

Citation		Cartilage endplate			
Author	Year	Fetal or infant (0 to 2)	Children and youth (2–25)	Adult (25 to 65)	Older adult (65+)
Eckert^18^	1947	—	• Channels are identified before third decade but otherwise avascular	• cCannels disappear leaving minute scars • Vascularisation in n = 2	• Vascularisation in n = 2
Hirsch^20^	1967	• Avascular except along the anterior edges where the osseous epiphyses are being formed (up to age 14)		• Reactive hyper vascularized tissue first detected • Degenerated endplates show irregular ingrowth of blood vessels through enlarged calcified zones	
Hassler^21^	1969	• Dense capillary network along the vertebral margin and several vessels penetrating into endplate	• Penetrating vessels were smaller and fewer in children (up to age 12)	• Penetrating vessels absent in adults • With degeneration, uneven vertebral margins of blood vessels and irregular secondary vascular ingrowths	
Donisch^22^	1971	• Numerous channels containing wide lumen with large blood vessels and capillaries	• Few blood vessels noted after age 2	—	—
Rudert^24^	1993	• Blood vessels penetrating were identified (up to age 7)		• Avascular after 30 to 39 years	
Stabler^25^	1996	—	—	• Contrast enhancement adjacent to vertebral endplates, associated with erosion of endplates (n = 14/21 enhanced IVDs); 0 controls	
Chandraraj^26^	1998	• Channels containing 2 to 3 thin‐walled endothelial tubes	• Increased bony tissue of channel walls • Avascular by 8 years	—	—
Boos^30^	2002	• Vascularized by thin‐walled blood vessels	• Regression in the number of physiologic blood vessels by 2 years, obliteration by 11 to 16 years	• Damage to endplate frequently results in edge neovascularization	
Nerlich^8^	2007	• Several blood vessels perforating the endplate towards other IVD tissue	• Focal obliteration of channels • Focal blood vessel invasion into damaged tissue		

Abbreviation: IVD, intervertebral disc.

**TABLE 4:** Vascularization of the annulus fibrosus

Citation		Annulus fibrosus			
Author	Year	Fetal or infant (0‐ 2)	Children and youth (2‐25)	Adult (25‐65)	Older adult (65+)
Eckert^18^	1947	—	• Tissue fibrosis and vascularization with age • Derived from vasculature to the longitudinal ligaments or adjacent loose areolar tissue		
Hirsch^19^	1953	• Blood vessels derived from perichondrium of developing tissues and penetrate toward marginal edge during ossification • Avascular after cessation of growth		• Avascular throughout adult life • Blood vessels to longitudinal ligaments do not penetrate tissue • Presence of highly vascular reactive tissue of damaged tissue	
Hirsch^20^	1967	• Avascular		• Marginal hyper vascular reactive tissue during degeneration, fissures, or clefts	
Hassler^21^	1969	• Richly vascularized • Derived from vasculature to the longitudinal ligaments	• Blood vessels were smaller and less prevalent with age	• Similar vascularization with young tissues • Uneven vascular ingrowths in damaged tissue	
Donisch^22^	1971	• Avascular, blood vessels through cartilage endplate do not penetrate tissue		—	—
Jamiolkowska^23^	1981	• Blood vessels identified along whole circumference in agreement with laminar fibers• Never detected within inner layers of tissue	• Presence of very fine blood vessels in all preparations with good filling of vessels • Only in lateral positions (ie, no blood vessels deep to thick bands of longitudinal ligaments) • Never detected within inner layers of tissue		
Rudert^24^	1993	• Numerous capillaries in the dorsolateral and ventrolateral portions up to 20 years		• Avascular beyond 30 to 39 years	
Kauppila^7^	1995	—	—	• Vascularity of anterolateral part of the tissue increased with severity of degeneration • Blood vessels were vertically oriented, likely collaterals between vertebra traveling through AF	
Stabler^25^	1996	—	—	• Focal contrast enhancement of the AF in 5/53 patients; 0 controls • histology confirmed focal vascularization of AF	
Repanti^27^	1998	• Numerous thin‐walled and dilated blood vessels in the outer layers of the tissue • Derived from vasculature to the longitudinal ligaments	• Fewer capillaries that are localized to outer layers of the tissue • Derived from longitudinal ligaments or surrounding connective tissue		• Blood vessels penetrated into deeper layers of the tissue compared to previous age group
Johnson^29^	2001	—	• Blood vessels associated with nerves in the outer 1 to 2 lamellae of the tissue, but found as deep as fifth lamellae • Thicker arterioles, venules, and capillaries • Increased dept of ingrowth with degeneration and fissures • Blood vessels were more abundant than nerves and no samples were avascular		—
Melrose^31^	2003	• Blood vessels localized to outer layers of AF • Small arterioles and venules	—	—	—
Johnson^32^	2007	—	• Blood vessels localized to outer layers of AF • More prevalent and deeper in degenerated tissue • No blood vessels in n = 2 of non‐pathological specimens		—
Nerlich^8^	2007	• Several blood vessels extending into inner and outer layers (until 2 years) • Capillaries between ligament insertion and outer layer of AF	• Only small capillary blood vessels in outer layers of AF derived from vasculature to the longitudinal ligaments • Blood vessels penetrated deeper in posterior compared to anterior • Inverse correlation of blood vessel penetration and age • Except degeneration/tears which were associated with ingrowth of small capillary buds		
Kokubo^33^	2008	—	—	• Invasion of small blood vessels into inner layers of spondylotic tissue	
Lama^35^	2013	–	–	• Minimal invasion of single‐layer endothelial cell capillaries in the inner and outer layers (n = 2/11 degenerated discs, also with radial fissures)	
Binch^36^	2015	• Blood vessels localized to inner and outer layers of AF • Greater prevalence of blood vessels with degeneration, localized to inner layers of AF • No blood vessels in nondegenerate tissue; blood vessels in degenerate tissue (n = 17/74)			
Lama^9^	2018	—	• Blood vessels only within ligamentous regions, surrounding the outer layer of tissue (scoliosis group)	• Blood vessels localized to the inner and outer layers of AF (n = 3/11 degenerative IVD) • Blood vessels were always near or within fissures (but not all fissures contained blood vessels) • Blood vessels more prevalent than nerves, occupied large areas, and grew deeper into tissue	

Abbreviations: AF, annulus fibrosus; IVD, intervertebral disc.

#### 3.3.1 Nucleus pulposus

There was a lack of conclusive evidence that blood vessels were detected within the nucleus pulposus at any age, irrespective of methodology used to detect vascular structures (Table 2). A recent study reported the presence of blood vessels along the nucleus pulposus‐annulus fibrosus transition zone and the “abnormal” localization of blood vessels within the inner IVD tissues associated with degeneration; however, the analysis of abnormal localization did not differentiate between the nucleus pulposus and inner annulus fibrosus.^36^

#### 3.3.2 Cartilage endplate

The cartilage endplate is a tissue of the IVD that is vascularized during fetal development and in infants, but transitions to an avascular tissue with age (Table 3). A common feature of the cartilage endplate was the presence of channels through the tissue. In fetal and infant tissues, blood vessels and capillaries were identified and localized within these channels. The source of these vessels is likely the richly vascularized vertebral endplate of the adjacent vertebra. Sometime between birth and 10 years of age there is a drastic decrease in the presence of blood vessels and capillaries within these channels, which are completely obliterated in adults.^8^, ^18^, ^30^ Focal obliteration of the cartilage endplate vessels was already identified in some infantile IVDs at 27 weeks to 2 years of age, as a possible first sign of vascular regression.^8^ In tissues from adults, it was noted that the cartilage endplates were free of blood vessels except for instances of focal damage that resulted in the invasion of blood vessels into the cartilage endplates.^18^, ^25^, ^30^

#### 3.3.3 Annulus fibrosus

In general, the annulus fibrosus is a vascularized tissue of the IVD but the localization of blood vessels is variable (Table 4). In fact, the spatial localization of blood vessels within the annulus fibrosus was poorly annotated. For example, two studies described the presence of blood vessels relative to concentric lamellae of the tissue^8^, ^29^ and another two studies provided a quantitative measure of distance from the periphery.^9^, ^23^ In general, early studies refer to the annulus fibrosus as a whole whereas more recent studies differentiate between the inner and outer layers of the tissue.

Nonetheless, the annulus fibrosus of fetal and infantile tissues was richly vascularized. Interestingly, one study using gross dissection reported blood vessels around the entire periphery of the annulus fibrosus in fetal and infantile tissues, and also reported blood vessels confined to the lateral positions of the IVD with age.^23^ Another article using histology reported an abundance of blood vessels in infantile tissues from 27 weeks to 2 years of age in the outer layers of the annulus fibrosus, extending toward the inner layers of the annulus fibrosus.^8^ The source artery or arteries supplying these blood vessels to the annulus fibrosus were not identified. Some authors noted the presence of blood vessels traveling through longitudinal ligaments and penetrating the outer layers of the annulus fibrosus.^8^, ^9^, ^18^, ^21^, ^27^ Others reported a lack of blood vessels associated with the longitudinal ligaments.^19^, ^23^, ^24^ Regardless, it is evident that blood vessels are present in the annulus fibrosus in fetal stages and in infants. Similar to the cartilage endplate, the detection of these blood vessels in the annulus fibrosus drastically decreases between 1 and 30 years of age.^8^, ^20^, ^21^, ^27^ In adults, the annulus fibrosus is less vascularized, yet blood vessels do persist in the outermost lamellar layers. With advancing age (>50 years of age) blood vessels were more often identified in the inner layers of the annulus fibrosus than in younger adults (25‐50 years of age), resembling the vascularization noted in fetal/infant tissues.^21^, ^27^ Importantly, damage (eg, degeneration or injury) to the annulus fibrosus was characterized by the invasion of blood vessels.^7^, ^18^, ^19^, ^20^, ^21^, ^32^ Therefore, it was common for IVDs from donors of advanced age with indicators of degeneration to show highly vascularized annulus fibrosus tissues.

Of note, a radiological and histological description of blood vessels in the lumbar IVD reported that vascular structures ran vertically through the annulus fibrosus.^7^ This is relevant for anastomotic connections between the vertebra as the authors also concluded that regression of these arteries preceded imaging signs of IVD degeneration.^7^

## 4 DISCUSSION

### 4.1 Summary of evidence

The objective of this comprehensive scoping review was to summarize evidence from the scientific literature related to the vascularization of the IVD in humans across the lifespan. Analysis of 22 articles identified through our comprehensive search revealed a lack of conclusive evidence of nucleus pulposus vascularization throughout life. Conversely, the presence and spatial localization of vasculature to the annulus fibrosus and cartilage endplates changes throughout the lifespan—with a rich blood supply in fetal and infantile tissues that decreases with age. It was evident that tissues of advanced age (often damaged or degenerated) were associated with the ingrowth of blood vessels deeper into the annulus fibrosus compared to age‐matched nondamaged tissues. As such, vascularization likely has important implications for the biology and pathobiology of the IVD.

Most of the articles were limited to analysis of the lumbar region and thus differences in vasculature of the IVD based on region of the vertebral column could not be discerned. While the source arteries likely differ between regions, tributary branches from the source arteries supplying other soft tissues of the vertebral column (eg, longitudinal ligaments) likely supply the periphery of the annulus fibrosus as well. However, studies are conflicted as to whether blood vessels are directly supplying the annulus fibrosus or dissociating from blood vessels supplying the longitudinal ligaments.^18^, ^19^, ^21^, ^23^, ^24^ In contrast, the blood vessels of the cartilage endplates clearly arise from the spongiosa of the associated vertebra, which is richly vascularized.^37^

Age‐associated changes to the extent of vasculature of the IVD were highlighted in this scoping review. In fetal and infantile tissues, the annulus fibrosus and cartilage endplates were richly vascularized. Between 1 and 30 years of age, the blood vessels of the annulus fibrosus and cartilage endplates were reduced or obliterated. It appears that these changes occur earlier in the cartilage endplate compared to the annulus fibrosus. It is paradoxical that the vascular supply of the IVD is diminishing during a time of rapid growth. Meanwhile, blood vessels within the outer annulus fibrosus are evident throughout the lifespan but are less abundant in adults compared to fetal and infantile IVDs. These findings may be related to several properties of the IVD, including that cell density is high in the outer annulus fibrosus, but low in the central tissues of the IVD. Alternatively, it may be that the vascular supply within the annulus fibrosus does not expand as the IVD grows, resulting in blood vessel penetration limited to the outer aspects of the tissue. Due to inconsistencies in the annotation of the spatial localization of blood vessels in the annulus fibrosus and the limitations of histology (eg, measured relative to numbered concentric lamellae, distance from the outer edge, or reported as outer vs inner layers), the current review was unable to report the exact localization or depth of vascularization in the annulus fibrosus, beyond indicating that it as limited to the periphery of the IVD. Of note, early studies did not delineate between the inner and outer layers of the annulus fibrosus, which is a more recent standard of IVD nomenclature marked by cells with distinct morphologies, gene, and extracellular matrix protein expression.^38^ Four articles reported the localization of vascularization of the IVD based on the distance of blood vessels from the periphery of the annulus fibrosus or the number of concentric lamellae reached.^8^, ^9^, ^23^, ^29^ Such methods of counting lamellae or measuring distance may not be reproducible based on differences in size and shape of IVDs across anatomical regions or between individuals and sexes. Moreover, referencing concentric lamellae may be difficult to apply to methods of contrast radiography where the soft tissues cannot be as easily differentiated, but may be applicable to magnetic resonance imaging investigations. Overall, the variability in the annotation of the spatial localization of blood vessels within the annulus fibrosus is currently limited to the broad‐stroke categorization of the outer layers of the annulus fibrosus.

A consistent finding within the literature was the prevalence of vascular ingrowths extending into the inner layers of the annulus fibrosus of IVDs with advanced age and degeneration.^7^, ^21^, ^32^ In fact, vascular ingrowth within damaged tissues of the annulus fibrosus and cartilage endplate was demonstrated irrespective of age (but was predominate in older adults). Others have proposed that annular fissures or tears associated with IVD degeneration are conducive to the ingrowth of blood vessels due to the focal loss of proteoglycans.^39^ These findings are supported by articles in the current review that demonstrated vascular ingrowths localized near or within damaged tissue, independent of age.^8^, ^9^, ^32^, ^36^ It is likely that blood vessel ingrowth is stimulated as a healing response due to the reduction of anti‐angiogenic factors (eg, proteoglycans) and the increased secretion of angiogenic growth factors and cytokines (eg, VEGF and IL‐1β).^40^ Moreover, within degenerative tissues, vascular ingrowths were often reported to be accompanied by neural ingrowths.^9^, ^29^, ^36^ Interestingly, both the presence of blood vessels without nerves^29^ and the presence of nerves without blood vessels^35^ were reported. Similarly, not all degenerated and damaged tissues were associated with vascular ingrowth. Ultimately, there is likely an interdependent relationship between neural and vascular ingrowth in IVD degeneration; however, it remains unclear whether vascular changes are a precursor to neural changes or vice versa. Clinically, elucidating the relationship between neural and vascular changes to the IVD in humans may have important implications related to the concept of discogenic back pain.

### 4.2 Limitations

There are several notable limitations of the evidence included in the review that should be considered in its collective interpretation. The challenge associated with accessing IVD tissues in humans limits the generalizability of the findings highlighted by this scoping review. Since tissues are obtained from surgery and postmortem evaluation, studies are often limited to small sample sizes and there is generally an overrepresentation of tissues retrieved from donors of advanced age with unknown comorbidities. Also, the variability and inconsistency in the annotation of study samples and vascular measurements did not provide the opportunity to identify sex‐related differences or to quantitatively map the spatial localization of blood vessels within IVD tissues. The development of consistent methodologies and nomenclature to describe and localize vasculature of the IVD is needed. In addition, the ability to characterize the spatial localization of blood vessels in IVD tissues was limited by the sensitivity of the methodologies used. For example, most studies used histology to evaluate the prevalence and localization of vasculature; which provides high‐resolution evaluation of tissues but is time consuming and limited to two‐dimensional analyses. Future studies using advanced imaging methods such as contrast enhanced microcomputed tomography or confocal microscopy with immunofluorescence may allow for an improved understanding of the three‐dimensional vasculature of the IVD, including potential source arteries. A limitation of the review itself is the exclusion of non‐English and inaccessible articles, which may have led to the loss of relevant evidence.

## 5 CONCLUSIONS

In summary, the current scoping review detailed the current state of knowledge of the vascularization of the IVD in humans. Histology is the predominate method to study blood vessels of the IVD, capitalizing on the specificity of immunohistochemistry markers of endothelial cells (eg, CD31, CD34). Overall, the IVD is not entirely avascular, as often cited. While it was confirmed that the nucleus pulposus is avascular throughout life, both the cartilage endplate and annulus fibrosus receive variable blood supplies across the lifespan, with the localization and prevalence of blood vessels varying by age and with tissue degeneration or damage. In fact, the annulus fibrosus should be thought of as a vascularized tissue and the question should be reframed as, “how vascularized is the annulus fibrosus and what are the clinical implications?”

## CONFLICT OF INTEREST

The authors declare no potential conflict of interest.

## AUTHOR CONTRIBUTIONS

All authors: Contributed substantially to the conception or design of the work, and the acquisition, analysis, or interpretation of data. Drafted and revised the work for important intellectual content. Approved the final version to be published. Agree to be accountable for all aspects of the work in ensuring that questions related to the accuracy or integrity of any part of the work are appropriately investigated and resolved.

## Supporting information


**Appendix** **S1:** Supporting information
