# Supplementary material for: Vascularization of the human intervertebral disc: A scoping review
Source: JOR Spine. 2020 Sep 15;3(4):e1123. doi: 10.1002/jsp2.1123 (PMC7770199; doi:10.1002/jsp2.1123)
Supplement: Supplementary file 1 — Appendix S1: Supporting information [file JSP2-3-e1123-s001.docx]

**Appendix S1: PubMed search strategy (performed on 2019-05-30)**

| **Concept** | **Search terms** | **Records**  **retrieved** |
| --- | --- | --- |
| #1  human | ("Infant, Newborn"[Mesh Terms] OR "Newborn infant"[All Fields] OR "Newborn infants"[All Fields] OR "newborns"[All Fields] OR "newborn"[All Fields] OR "neonates"[All Fields] OR "neonate"[All Fields] OR "Infant"[Mesh Terms] OR "infant"[All Fields] OR "infants"[All Fields] OR "Child"[Mesh Terms] OR "children"[All Fields] OR "Child, Preschool"[Mesh Terms] OR "preschool child"[All Fields] OR "preschool children"[All Fields] OR "Adolescent"[Mesh Terms] OR "adolescents"[All Fields] OR "adolescence"[All Fields] OR "teens"[All Fields] OR "teen"[All Fields] OR "teenagers"[All Fields] OR "teenager"[All Fields] OR "youth"[All Fields] OR "youths"[All Fields] OR "Young adult"[Mesh Terms] OR "youth adult"[All Fields] OR "young adults"[All Fields] OR "Adult"[Mesh Terms] OR "adults"[All Fields] OR "Middle Aged"[Mesh Terms] OR "middle aged"[All Fields] OR "middle age"[All Fields] OR "Aged"[Mesh Terms] OR "elderly"[All Fields] OR "Aged, 80 and over"[Mesh Terms] OR "nonagenarians"[All Fields] OR "nonagenarian"[All Fields] OR "octogenarians"[All Fields] OR "octogenarian"[All Fields] OR "centenarians"[All Fields] OR "centenarian"[All Fields] OR "humans"[MeSH Terms] OR "humans"[All Fields] OR "human"[All Fields] OR "homo sapien"[All Fields] OR "homo sapiens"[All Fields] OR "male"[All Fields] OR "female"[All Fields] OR "male"[MeSH Terms] OR "female"[MeSH Terms] OR "cadaver"[MeSH Terms] OR "cadaver"[All Fields] OR "cadavers"[All Fields] OR "autopsy"[MeSH Terms] OR "autopsy"[All Fields] OR "autopsies"[All Fields] OR "post-mortem examination"[All Fields] OR "post mortem examination"[All Fields] OR "post mortem examinations"[All Fields] OR "post-mortem examinations"[All Fields] OR "postmortem examination"[All Fields] OR "postmortem examinations"[All Fields]) | 20,989,252 |
| #2  intervertebral  disc | ("intervertebral disc"[MeSH Terms] OR "intervertebral disc"[All Fields] OR "intervertebral discs"[All Fields] OR "intervertebral disk"[All Fields] OR "intervertebral disks"[All Fields] OR "vertebral disc"[All Fields] OR "vertebral discs"[All Fields] OR "vertebral disk"[All Fields] OR "vertebral disks"[All Fields] OR "spinal disc"[All Fields] OR "spinal discs"[All Fields] OR "spinal disk"[All Fields] OR "spinal disks"[All Fields] OR "anulus fibrosus"[All Fields] OR "annulus fibrosus"[All Fields] OR "anulus fibrosis"[All Fields] OR "annulus fibrosis"[All Fields] OR "annulus fibrosus"[MeSH Terms] OR "Nucleus Pulposus"[Mesh Terms] OR "nucleus pulposus"[All Fields]) | 35,457 |
| #3  vascular | ("neovascularization, pathologic"[MeSH Terms] OR "pathologic neovascularization"[All Fields] OR "pathological neovascularization"[All Fields] OR "pathologic angiogenesis"[All Fields] OR "vascularization"[All Fields] OR "blood supply"[All Fields] OR "blood vessels"[MeSH Terms] OR "blood vessels"[All Fields] OR "blood vessel"[All Fields] OR "angiography"[MeSH Terms] OR "angiography"[All Fields] OR "angiographies"[All Fields] OR "angiogram"[All Fields] OR "angiograms"[All Fields] OR "arteriography"[All Fields] OR "arteriographies"[All Fields] OR "microradiography"[MeSH Terms] OR "microradiography"[All Fields] OR "microradiographies"[All Fields] OR "arteries"[MeSH Terms] OR "arteries"[All Fields] OR "artery"[All Fields] OR "capillaries"[MeSH Terms] OR "capillary"[All Fields] OR "capillaries"[All Fields]) | 1,591,377 |
| #4 | #1 AND #2 AND #3 | 1344 |
